# *OsbHLH073* Negatively Regulates Internode Elongation and Plant Height by Modulating GA Homeostasis in Rice

**DOI:** 10.3390/plants9040547

**Published:** 2020-04-23

**Authors:** Jinwon Lee, Sunok Moon, Seonghoe Jang, Sichul Lee, Gynheung An, Ki-Hong Jung, Soon Ki Park

**Affiliations:** 1 School of Applied Biosciences, Kyungpook National University, Daegu 41566, Korea; leejinwon@outlook.com; 2 Graduate School of Biotechnology & Crop Biotech Institute, Kyung Hee University, Yongin 17104, Korea; moonsun@khu.ac.kr (S.M.); genean@khu.ac.kr (G.A.); 3 World Vegetable Center Korea Office (W.K.O), Jellabuk-do 55365, Korea; seonghoe.jang@worldveg.org; 4 Center for Plant Aging Research, Institute for Basic Science (IBS), Daegu 42988, Korea; scironlee@gmail.com

**Keywords:** bHLH transcription factor, gibberellin, GA homeostasis, internode elongation, OsbHLH073, plant height, rice

## Abstract

Internode elongation is one of the key agronomic traits determining a plant’s height and biomass. However, our understanding of the molecular mechanisms controlling internode elongation is still limited in crop plant species. Here, we report the functional identification of an atypical basic helix-loop-helix transcription factor (*OsbHLH073*) through gain-of-function studies using overexpression (*OsbHLH073-OX*) and activation tagging (*osbhlh073-D*) lines of rice. The expression of *OsbHLH073* was significantly increased in the *osbhlh073-D* line. The phenotype of *osbhlh073-D* showed semi-dwarfism due to deficient elongation of the first internode and poor panicle exsertion. Transgenic lines overexpressing *OsbHLH073* confirmed the phenotype of the *osbhlh073-D* line. Exogenous gibberellic acid (GA_3_) treatment recovered the semi-dwarf phenotype of *osbhlh073-D* plants at the seedling stage. In addition, quantitative expression analysis of genes involving in GA biosynthetic and signaling pathway revealed that the transcripts of rice *ent-kaurene oxidases 1* and *2* (*OsKO1* and *OsKO2*) encoding the GA biosynthetic enzyme were significantly downregulated in *osbhlh073-D* and *OsbHLH073-OX* lines. Yeast two-hybrid and localization assays showed that the OsbHLH073 protein is a nuclear localized-transcriptional activator. We report that *OsbHLH073* participates in regulating plant height, internode elongation, and panicle exsertion by regulating GA biosynthesis associated with the *OsKO1* and *OsKO2* genes.

## 1. Introduction

Gibberellin (GA) plays pivotal roles in many developmental processes including seed germination, root growth, stem and hypocotyl elongation, the promotion of cell division and elongation, flower induction, and internode elongation [1,2,3,4]. The GA biosynthetic pathway has been analyzed in detail in genetic and chemical studies in higher plants [4,5,6]. In higher plants, GA biosynthesis can generally be divided into three subcellular compartmentalization: Plastids, the endoplasmic reticulum (ER) membrane, and the cytoplasm. In plastids, two enzymes, ent-copalyl diphosphate synthase (CPS) and ent-kaurene synthase (KS) are converted from geranylgeranyl diphosphate to ent-kaurene through a two-step cyclization [7,8]. Two cytochrome p450 monooxygenases, ent-kaurene oxidase (KO) and ent-kaurenoic acid oxidase (KAO), convert ent-kaurene into GA12 [8,9,10,11]. GA13 oxidase converts GA12 into GA53 [12]. In the cytoplasm, GA 20-oxidase (GA20ox) and GA 3-oxidase (GA3ox) convert from GA12 and GA53 to various GA intermediates (GA44, GA19, and GA20) and bioactive GAs (GA1, GA3, and GA4), respectively [13,14]. GA 2-oxidase deactivates active GAs [15,16,17,18,19].

Semi-dwarfism is a very important trait for breeding cereal crops because it is associated with improved lodging resistance and a very good harvest index [14,20,21]. Mutations of *GA20ox2* (*sd1*) and *GA3ox2* (*d18*) display a loss of function, resulting in dwarfism in rice [16]. The overexpression of *GA2-oxidase* (*GA2ox*) genes in Arabidopsis, rice, and other plants also results in a dwarf phenotype [18,22,23,24]. The *TOMATO INTERNODE ELONGATED1-1(TIE1-1)* and *ELONGATED INTERNODE (EI)* mutants display internode elongation in tomato. *TIE1-1/EI* encodes a class III GA 2-oxidase, a GA2oxidase 7 [25,26]. The overexpression of the *ELONGATED UPPERMOST INTERNODE1* (*EUI1*) gene causes the dwarf phenotype, and Null mutations of the *EUI1* gene result in the accumulation of active GAs and elongated uppermost internodes and increased plant height [2,3,12,27]. A class I homeodomain-leucine zipper, *HOX12*, positively controls the expression of *EUI1* by directly binding to the promoter region [28]. In addition, a C2H2 zinc finger, *PREMATURE INTERNODE ELONGATION 1*, plays a negative role in internode elongation in rice [29].

Basic helix-loop-helix (bHLH) transcription factors (TFs) play diverse roles in controlling various biological processes in both plants and in animals. In rice, there are 177 bHLH genes, while there are 183 in poplar, 167 in Arabidopsis, and 98 in moss [30]. The bHLH domain consists of 60 amino acids organized in a basic region and an HLH region. The basic region is needed for DNA binding and the HLH domain is needed for protein–protein interaction [31]. bHLH TFs are divided into two groups according to their DNA-binding activity, the atypical non-DNA-binding and the typical DNA-binding bHLH family [32]. Both atypical and typical bHLH TFs regulate various biological processes via protein–protein interaction. For example, in Arabidopsis, *PHYTOCHROME INTERACTING FACTORS 4* and *5* (*PIF4* and *PIF5*), encoding typical DNA-binding bHLH TFs, function in light signaling and hypocotyl growth. It has recently been reported that *PIF3, PIF4, PIF5*, and *PIF3-LIKE 5 (PIL5)* are involved in the GA biosynthesis and signaling pathway in Arabidopsis [4]. An atypical bHLH TF, *LONG HYPOCOTYL IN FAR-RED1*, modulates phytochrome signaling via heterodimerization with *PIF4* and *PIF5* [33]. In rice, typical bHLH TFs are involved in the development of the tapetum, internode elongation, grain size, iron homeostasis, and hormone signaling. Thus, *UNDEVELOPED TAPETUM1* is needed for early tapetum development in rice [34], and overexpression of *OsPIL1/OsPIL13* increases internode cell size and promotes internode elongation in rice [35]. *POSITIVE REGULATOR OF GRAIN LENGTH1, 2* (*PGL1, 2*) and *ANTAGONIST OF PGL1* (*APG*) are implicated in the processes determining grain length and weight in rice [36,37]. *OsbHLH107* and its homolog, *OsPIL11*, regulates the grain size [38]. *OsbHLH057* and *OsbHLH058* positively regulated the iron deficiency responses [39]. Atypical bHLH TFs, *INCREASED LAMINAR INCLINATION* (*ILI*) and *ILI1 BINDING bHLH1 (IBH1),* regulate cell elongation and lamina joint bending in rice [40]. A complex consisting of *BRASSINOSTEROID (BR) UPREGULATED1-LIKE1 (OsBUL1)* and *OsBUL1 COMPLEX1 (OsBC1)* regulates leaf angle and grain size [41], and *LAX PANICLE (LAX)* is needed for the initiation and maintenance of axillary meristems in rice panicle [42]. *OsbHLH035* involved in the seed germination through ABA-dependent and independent manners, respectively [43].

In rice, although some atypical bHLH TFs are involved in hormone signaling [41,44,45], shoot branching [46], and the determination of grain length and weight [36,37], only a few atypical bHLH genes have been functionally identified. In this study, we identified and characterized an atypical bHLH TF, *OsbHLH073*, that negatively regulates plant height by suppressing internode elongation. Yeast two-hybrid and protoplast assays suggest that *OsbHLH073* has a role as a nuclear localized-transcriptional activator. In addition, expression analysis of the GA biosynthetic genes in the activation and overexpression lines of *OsbHLH073* proposes the molecular mechanism on the function of *OsbHLH073* associated with GA biosynthesis.

## 2. Results

### 2.1. Isolation of a Semi-Dwarf Phenotype Mutant by Simulating OsbHLH073

**Figure 1:**
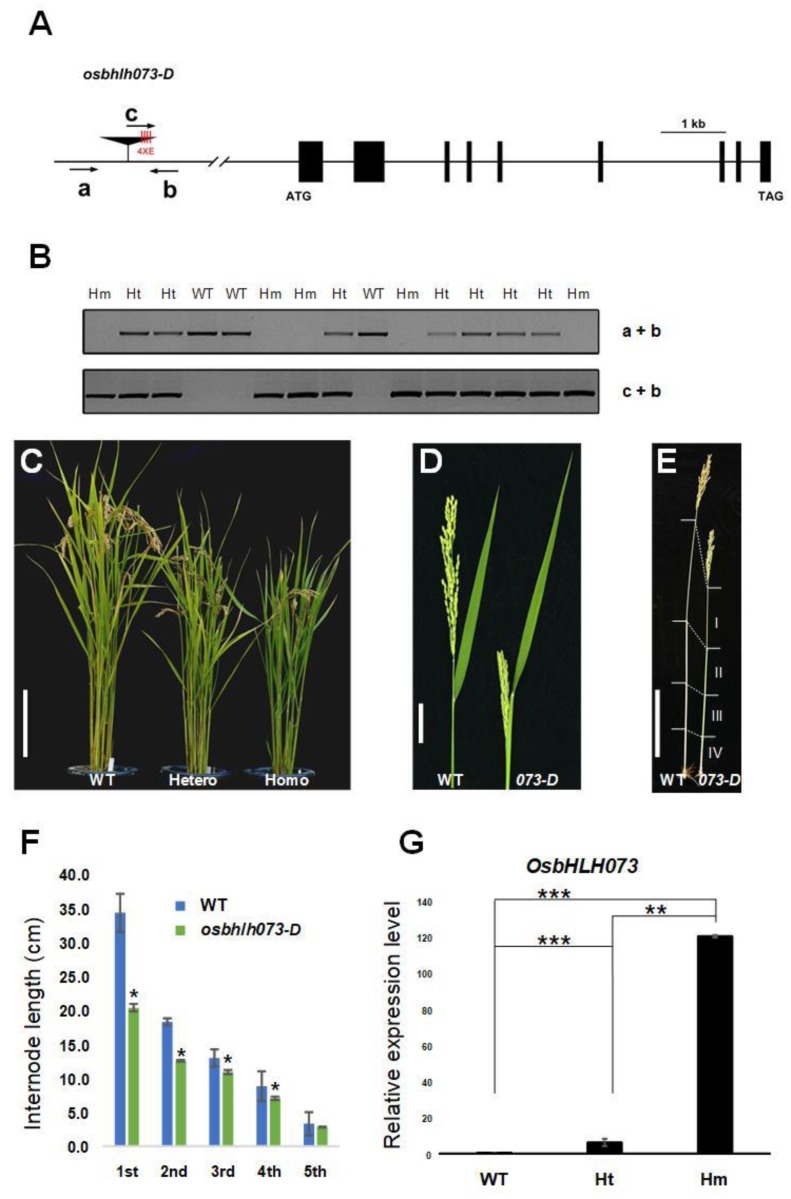
Phenotype of *osbhlh073-D* plants and the expression level of *OsbHLH073.* (**A**) Schematic diagram of the *OsbHLH073* gene structure and relative T-DNA insertion positions. Black boxes denote exons. 4XE denotes four tandem copies of *CaMV 35S* enhancer. Primers (a, b, and c) used for genotyping. (**B**) Genotyping analysis of *osbhlh073-D* plants. WT, Ht, and Hm mean wild-type segregant, heterozygote, and homozygote for T-DNA insertion, respectively. (**C**) Phenotype of wild-type, heterozygous, and homozygous plants. Bar = 20 cm. (**D**) Panicle exsertion phenotype of wild-type and o*sbhlh073-D* (*073-D*) plants. Bar = 5 cm. (**E**) Photograph showing internode length of wild-type and *osbhlh073-D* (*073-D)* plants. Bar = 20 cm. (**F**) Average internode length of wild-type and o*sbhlh073-D* plants. Data are mean ± standard deviation (SD) from at least 10 plants. (* *p* < 0.001, Student’s *t*-test) (**G**) Relative expression level of *OsbHLH073* in wild-type, heterozygous, and homozygous plants. Expression levels were normalized to *Actin* (*LOC_Os03g50885*) (** *p* < 0.01, *** *p* < 0.001, Student’s *t*-test).

We isolated a semi-dwarf phenotype mutant line, PFG_4A-02508, from our T-DNA tagging population [47,48,49]. Flanking sequence analysis revealed that T-DNA was inserted 8489 bp upstream of the start codon of *LOC_Os05g14010* on chromosome 5 (Figure 1A). The gene encodes *OsbHLH073*, an atypical bHLH TF of the predicted 177 bHLH genes of rice (Figure S1) [30,32]. Segregation and genotyping analysis of the PFG_4A-02508 plants showed dominant semi-dwarf phenotypes observed in heterozygous and homozygous progeny. Collectively, these results indicate that the phenotype is likely caused by gain-of-function mutations (Figure 1B,C). qRT-PCR analysis displayed that the transcript of *OsbHLH073* was significantly increased in all heterozygous and homozygous plants (Figure 1G). We named this line *osbhlh073-D*.

**Figure 2:**
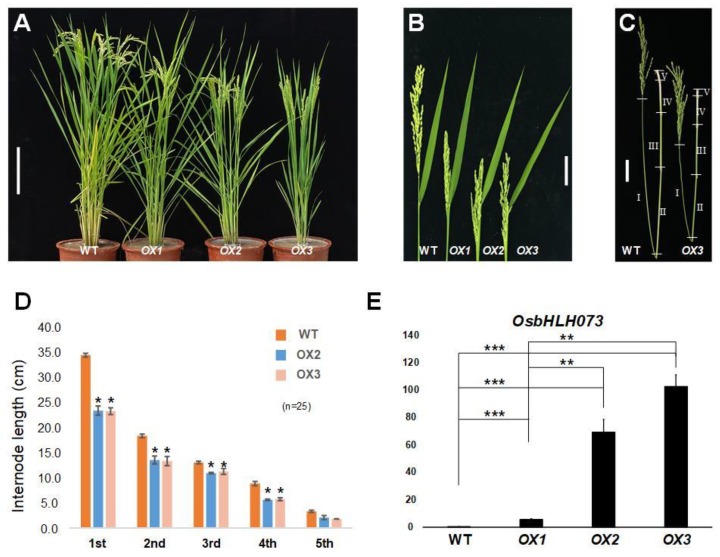
Phenotype of *OsbHLH073*-*overexpression* (*OX*) plants and the expression levels of *OsbHLH073.* (**A**) Phenotype of wild-type and *OsbHLH073-OX* plants. Bar = 20 cm. (**B**) Panicle exsertion phenotype of wild-type and *OsbHLH073-OX* plants. Bar = 5 cm. (**C**) Photograph showing internode length of wild-type and *OsbHLH073-OX* plant. Bar = 5 cm. (**D**) Average internode length of wild-type and *OsbHLH073-OX3* plant. Data are mean ± SD from 25 plants (* *p* < 0.001, Student’s *t*-test). (**E**) Relative expression level of *OsbHLH073* in wild-type and *OsbHLH073-OX* plants. Expression levels were normalized to *OsActin* (*LOC_Os03g50885*) (** *p* < 0.01, *** *p* < 0.001, Student’s *t*-test).

At the reproductive stage, *osbhlh073-D* plants showed the defective panicle exsertion phenotype besides a semi-dwarf compared to wild-type (Figure 1C,D). The poor panicle exsertion was mostly caused by inhibition of the elongation of the first and second internodes, resulting in the failure of the panicles of *osbhlh073-D* to fully emerge from the leaf sheath (Figure 1E,F). To confirm mutant phenotypes, we generated *OsbHLH073*-overexpressing (OX) plants constitutively expressing *OsbHLH073* under the control of the maize ubiquitin promoter via *Agrobacterium*-mediated transformation in a Dongjin rice background [50]. We selected three lines among 6 T_0_ plants. Those selected lines were used for the generation of T2 homozygous lines and all displayed the similar phenotype to the *osbhlh073-D* mutant (Figure 2A–D). We also confirmed that the mRNA level of *OsbHLH073* in the T_2_ OX plants (OX1- OX3) was correlated to the semi-dwarf phenotype (Figure 2A,E). This result indicates that *OsbHLH073* regulates internode elongation in a negative way.

### 2.2. OsbHLH073 and OsbHLH074 Knockout Mutants Have no Visible Phenotype

**Figure 3:**
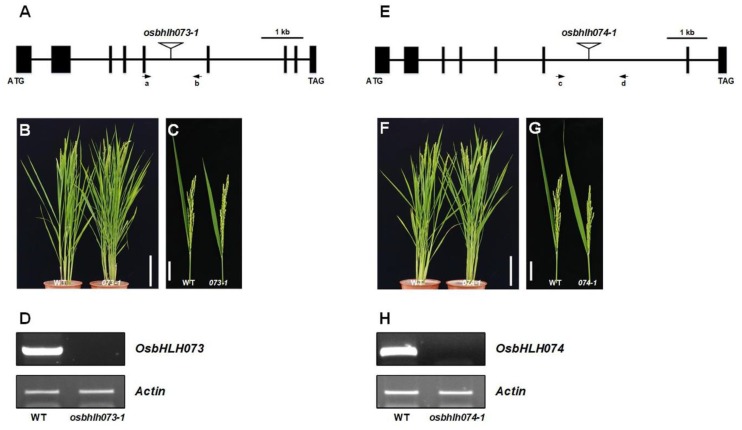
Phenotypes of *osbhlh073-1* and *osbhlh074-1* mutants. (**A**) Genomic structure of *OsbHLH073* and the positions of T-DNA insertion. Black boxes denote exons. Primers (a, b) were used for genotyping. (**B**) Phenotype of wild-type and *osbhlh073-1(073-1)* plants. (**C**) Panicle exsertion phenotype of wild-type and *073-1* mutant. Bar = 5 cm. (**D**) RT-PCR analyses of *OsbHLH073* transcripts in wild type (WT) and *073-1* mutant. (**E**) Scheme diagram of the *OsbHLH074* gene and the T-DNA insertion positions. Black boxes denote exons. Primers (c, d) were used for genotyping. (**F**) Phenotype of wild-type and *osbhlh074-1(074-1)* plants. Bar = 20 cm. (**G**) Panicle exsertion phenotype of wild-type and *074-1* mutant. Bar = 5 cm. (**H**) RT-PCR analyses of *OsbHLH074* transcripts in wild-type (WT) and *074-1* mutant. *Actin* (*LOC_Os03g50885*) was used as the control.

To investigate the function of *OsbHLH073*, we screened T-DNA insertional mutant pools and selected a mutant in which T-DNA was inserted into the fifth intron of *OsbHLH073* (PFG_3A-17056) (Figure 3A). *OsbHLH073* transcript was not detected in this line, indicating that *osbhlh073-1* is a knockout mutant of this gene (Figure 3D). At the heading stage, we examined the phenotype of the *osbHLH073-1* mutant, but did not observe any visible differences in phenotype (Figure 3B,C). Plant height and panicle exsertion were almost the same as in the wild-type. One possible explanation for this result could be that paralogous genes of O*sbHLH073* gene might result in functional redundancy. Phylogenetic analysis showed that two homologous genes, O*sbHLH072* and O*sbHLH074*, were present in the rice genome (Figure S2) [51]. The OsbHLH073 and OsbHLH074 protein sequences were 52% identical and had 61% similarity over the full length of the protein sequence, respectively, while the OsbHLH073 and OsbHLH072 protein had 41% identity and 51% similarity, respectively (Figure S3). Since *OsbHLH074* was revealed as the closest homologue of *OsbHLH073*, we isolated a T-DNA insertional mutant of *OsbHLH074* (PFG_5A-00405). T-DNA was inserted at the sixth intron of *OsbHLH074* (*LOC_Os01g13000*) on chromosome 1 (Figure 3E). Intact *OsbHLH074* transcript was not detectable in this mutant, indicating that *osbhlh074-1* is also a null mutant of this gene (Figure 3H). However, *osbhlh074-1* plant also did not show any visible phenotype, and the height and panicle exsertion of *osbhlh074-1* were quite similar to those of wild-type at the heading stage (Figure 3F,G). Since there were no obvious phenotypic alterations in each *osbhlh073* and *osbhlh074* mutant, genetic redundancy can be considered for explaining the results based on their sequence similarity.

### 2.3. Exogenous GA_3_ Application Rescues the Dwarf Phenotype of Osbhlh073-D Plants

**Figure 4:**
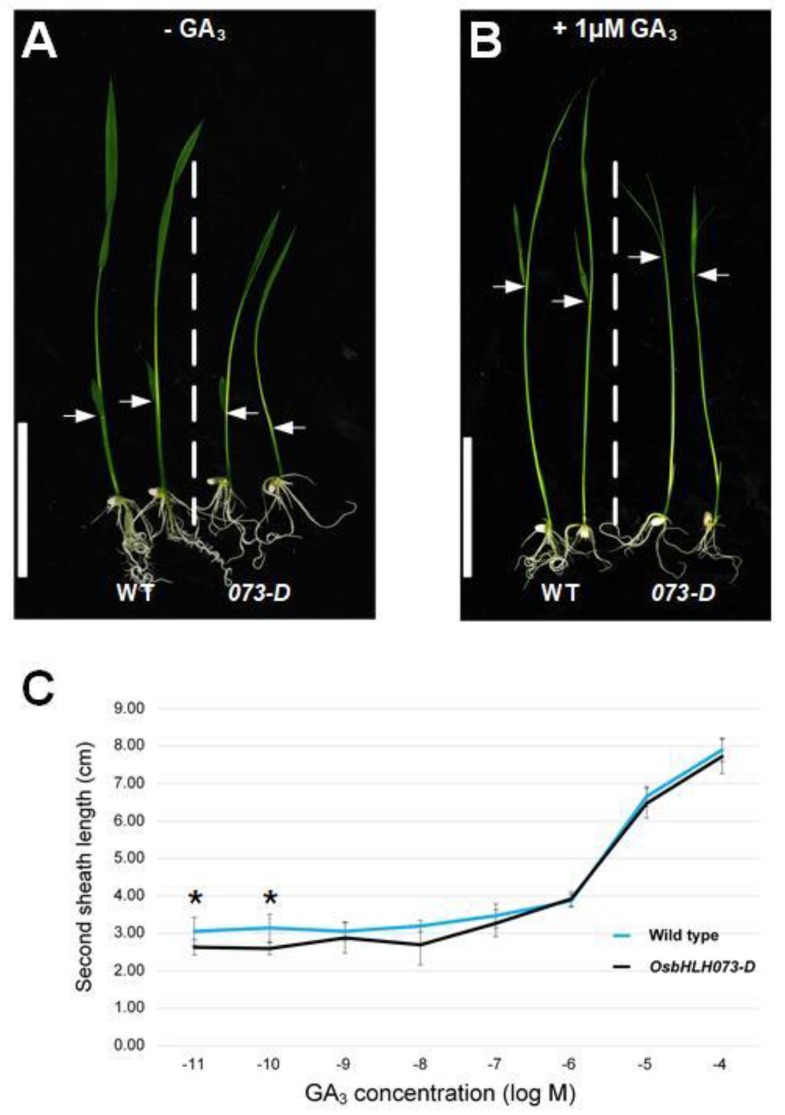
Rescue of the *osbhlh073-D* semi-dwarf phenotype by exogenous gibberellic acid (GA_3)_ application. (**A**) Phenotype of seven-day-old plants in the light without GA_3_. Arrow indicates the second leaf sheath. (**B**) Phenotype of seven-day-old plants in the light with 1 µM of GA_3_ added to the medium. Bar = 5 cm. Arrow indicates the second leaf sheath. (**C**) Measurement of second leaf sheath length after GA_3_ treatment. Data are mean ± SD from at least 10 plants. (* *p* < 0.05, Student’s *t* test).

In plants, various hormones are associated with plant height [20,52]. Among these hormones, GAs are very important factors in determining plant height [14,20,53]. At the reproductive stage, o*sbhlh073-D* showed a semi-dwarf phenotype. To test whether the semi-dwarf phenotype of *osbhlh073-D* was caused by insensitivity to or deficiency of GA, we measured the height (from the shoot base to the top leaf tip) (Figure 4A,B), and analyzed the response to exogenous GA_3_ application, and measured the second leaf sheaths of both wild-type and mutant growing on 1/2 MS median containing from 10^−11^ to 10^−4^ M GA_3_ in the continuous light condition for seven days (Figure 4C) [54]. Although *osbhlh073-D* showed a semi-dwarf phenotype compared to wild-type (Figure 4A), the response to application with GA_3_ in the length of the second leaf sheath of both wild-type and mutant was quite similar (Figure 4B,C). These results indicate that exogenous GA_3_ rescues the semi-dwarf phenotype of o*sbhlh073-D*, indicating that *osbhlh073-D* may be a GA-deficient mutant. This observation is consistent with results in other GA-deficient mutants in rice [1,16,28,55].

### 2.4. OsbHLH073 is Preferentially Expressed in Developing Organs at Both the Seedling and Reproductive Stages

**Figure 5:**
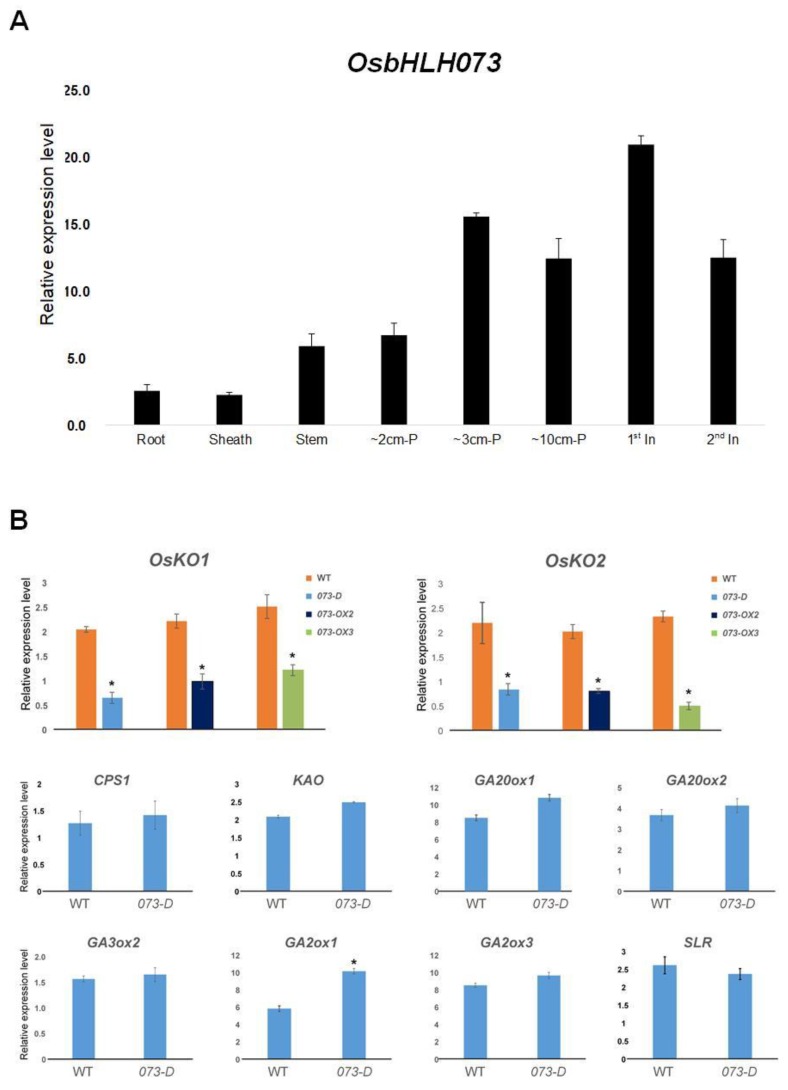
Expression patterns of *OsbHLH073* and GA metabolic genes. (**A**) Expression patterns of *OsbHLH073* in various organs. Fourteen-day-old plants were used for the root, sheath, and stems. Stem: Shoot base (around 2 cm); P: Panicle; In: Internode. (**B**) Expression pattern of *OsKO1*, *OsKO2*, and other GA biosynthetic and signaling pathway genes using mature panicles in o*sbhlh073-D* (*073-D*) and *OsbHLH073-OX* (*073-OX2, 073-OX3*) plants, respectively. Expression levels were normalized to *OsActin* (*LOC_Os03g50885*). (* *p* < 0.05, Student’s *t*-test).

To analyze the biological roles of *OsbHLH073*, we examined the *OsbHLH073* transcripts in various organs using real-time PCR. The various organs included the root, shoot base, and leaf sheath in 14-day-old plants, young panicles, the first four internodes, and mature panicles. At the seedling stage, we found that *OsbHLH073* transcripts were more abundantly expressed in the shoot base than in the root or leaf blade. At the reproductive stage, its expression was more abundant in the young panicle and the first internode than in the mature panicle and the second internode (Figure 5A). This observation is consistent with reports of many genes involved in GA biosynthesis in rice. For example, *EUI1* transcripts are mainly expressed in the young seedling shoot base and young panicles [2,3], and *OsKO1* transcripts are abundant in young panicles and elongated stems [56].

### 2.5. osbhlh073-D Alters the Expression of KO1 and KO2

It is well known that genes involved in GA biosynthesis and catabolism are negatively and positively regulated by active GAs [6]. To investigate the functional involvement of *OsbHLH073* in the GA biosynthetic or signaling pathways, we monitored the relative expression levels of GA biosynthesis-related genes using mature panicles of o*sbhlh073-D* and *OsbHLH073-OX* plants. As shown in Figure 5, the *OsKO1* and *OsKO2* transcripts were decreased in the *osbhlh073-D* and *OsbHLH073*-OX lines compared to wild-type (Figure 5B). The expression of gibberellin deactivation enzyme, GA2ox1, was increased in the o*sbhlh073-D* mutant (Figure 5B). However, the relative expression levels of other GA biosynthetic pathway-related genes did not show a significant difference between o*sbhlh073-D* and wild-type plants (Figure 5B). We also monitored the expression patterns of gene in the GA signaling pathway, and found that the expression of a negative regulator of gibberellin signaling, DELLA protein *SLR1* was not significantly different from that in wild-type plants (Figure 5B) [56].

Previously, it was shown that an *OsKO2*-weak allele mutant (*d35^Tan-Ginbozu^*) exhibited defective panicle exsertion and the semi-dwarf phenotype, and that knocking out *OsKO2* resulted in a more severe dwarf phenotype [1]. The phenotype of *d35^Tan-Ginbozu^* is quite similar to that of *osbhlh073-D* (Figure 1B–E). This result indicates that *OsbHLH073* may control the expression of the *OsKO1* and *OsKO2* genes to regulate internode elongation and GA homeostasis.

### 2.6. OsbHLH073 is a Nuclear Localized-Transcription Activator

**Figure 6:**
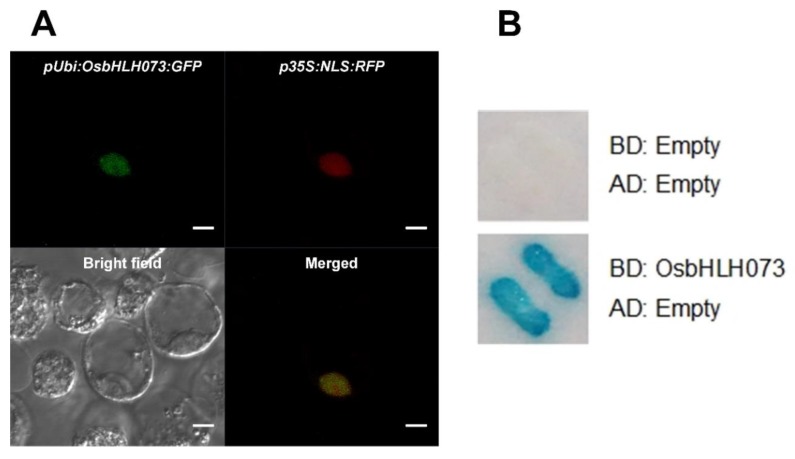
Localization of *OsbHLH073::GFP* protein in the nucleus and transcriptional activation assay. (**A**) Rice protoplast was co-transfected with *35S::NLS-mRFP* (positive control) and *pUbi::OsbHLHL073-GFP*. Bar = 5 μm. (**B**) *OsbHLH073* displays transcriptional activity in yeast. An empty vector was used as the control. GAL4-DNA binding domain and transcription activation domain for yeast two-hybrid system are presented as BD and AD, respectively. *OsbHLH073* was fused to the BD to generate *BD:OsbHLH073* fusion protein.

Most of the bHLH proteins are TFs. Some bHLH TFs, such as *AtbHLH112* [57] and *OsBC1*, are activators [41], whereas some others, such as *JA-ASSOCIATED MYC2-LIKE2 (JAM2)* and *JAM3* [58], are repressors. To investigate the function of *OsbHLH073*, we constructed the *OsbHLH073-GFP* fusion construct under the maize *ubiquitin1* promoter and transfected it into a rice protoplast. To control the localization in the nucleus, *35S::NLS-mRFP* vector was transfected into the rice protoplast. As expected, *NLS-mRFP* was localized in the nucleus and the *OsbHLH073-GFP* fusion protein was also detected in the nucleus (Figure 6A). We then examined the transcriptional activity of *OsbHLH073* using the yeast two-hybrid system [41]. As shown in Figure 6B, *OsbHLH073* showed transcriptional activation activity in yeast, even though it was not likely to contain a DNA-binding region.

## 3. Discussion

In this study, the function of *OsbHLH073* gene was investigated through gain-of-function approaches by activation tagging and overexpression analysis. We obtained and generated two classes of mutants (*osbhlh073-D* and *OsbHLH073*-*OX*, respectively), both of which displayed a semi-dwarf phenotype at the seedling stage, and poor panicle exsertion due to a defect in the elongation of the first and second internodes at the reproductive stage. Exogenous GA_3_ application rescued the dwarf phenotype of o*sbhlh073-D* plants at the seedling stage, indicating that *OsbHLH073* is involved in the GA biosynthetic pathway. However, there are no reports elucidating the functions of atypical bHLH TFs involved in GA signaling or biosynthesis pathways in rice. In Arabidopsis, *PHYTOCHROME-INTERACTING FACTOR 1* (*PIF1*), a typical bHLH TF, regulates GA responsiveness by directly binding to the promoter regions of *GIBBERELLIC-ACID INSENSITIVE* (*GAI*) and *REPRESSOR OF THE GAI* during seed development or germination [59]. Many bHLH TFs including *PIF1* have a conserved DNA-binding site, the E-box (5′-CANNTG-3′), to regulate cell-type-specific and developmental expression. *OsbHLH073* belongs to the atypical bHLH TFs without DNA-binding motif, but this bHLH displays transcriptional activity as a nuclear protein. Thus, the function of *OsbHLH073* might require the interaction with typical bHLHs like Arabidopsis *PIF1*.

The expression patterns of genes in the GA biosynthesis or signaling pathways revealed that both *OsKO1* and *OsKO2* genes were downregulated in the mature panicle of the *osbhlh073-D* and *OsbHLH073*-OX lines. From these findings, we propose that, at least in part, the *OsbHLH073* gene regulates plant growth and participates in GA homeostasis via regulation of the *OsKO1* and *OsKO2* genes. However, we did not perform the experiment to test a direct interaction of OsbHLH073 with the *OsKO1* and *OsKO2* promoter regions because OsbHLH073 belongs to the non-DNA-binding bHLH family, reflecting the existence of potential OsbHLH073-interacting TFs. T-DNA insertion mutants of *OsbHLH073* and *OsbHLH074*, the closest homolog to *OsbHLH073*, did not show clear visible phenotype, indicating that *OsbHLH073* and *OsbHLH074* can be functionally redundant. Investigating the phenotype by making a double mutant and observing the variant phenotype may give a better understanding on the function of these genes. Functional redundancy is very general in this gene family [60]. For example, the *pif1pif3pif4pif5* quadruple mutant displays a strong pleiotropic phenotype associated with constitutive-photomorphogenesis in the dark [61], and the triple mutant of *BR ENHANCED EXPRESSION 1, 2, AND 3 (BEE1, BEE2,* and *BEE3)* shows reduced hypocotyl elongation phenotype in response to simulated shade [44].

Previous studies have shown that the regulation of *KO2* expression by directly binding to the *KO2* promoter leads to alteration of the GA level and causes the dwarf phenotype. Tobacco transcription factor, *REPRESSION OF SHOOT GROWTH,* activates the expression of *KO2* gene in Arabidopsis [62]. *GIBBERELLIN-DEFICIENT DWARF1*/*BRITTLE CULM 12* directly binds to the promoter of *OsKO2* and causes the impaired cell elongation with dwarf phenotype [63]. *O. SATIVA LSD-ONE-LIKE 1* (*OsLOL1*; C2C2-type zinc finger protein) and *OsbZIP58* activates *OsKO2*, thereby inducing seed germination by affecting GA biosynthesis [64]. Rice *NAC DOMAIN-CONTAINING PROTEIN* 2 (*OsNAC2)* overexpression reduces plant height and internode length, and *OsNAC2* represses the expression of *OsKO2* by directly binding to its promoter, as shown in Chip-seq analysis and yeast one-hybrid analysis [65]. *OsbZIP48* directly binds to the promoter of *OsKO2* and regulates its expression to control internode elongation [66].

To identify how to regulate *OsKO1* and *OsKO2* gene expression and control the growth and GA homeostasis associated with *OsbHLH073*, it will be necessary to identify the proteins interacting with OsbHLH073. Previous studies reported that atypical bHLH proteins interact with typical bHLH TFs, which control the downstream genes [33,67,68,69]. For example, the Arabidopsis atypical bHLH TFs *PACLOBUTRAZOL RESISTANCE1* and *ILI1 BINDING bHLH PROTEIN1 (IBH1)* interact with each other and regulate the expression of many genes antagonistically by the interaction of IBH1 with the DNA-binding bHLH factor, *HOMOLOG OF BEE2 INTERACTING WITH IBH1* [68]. We manually analyzed the promoter sequences of *OsKO1* and *OsKO2* and revealed that there were three and nine conserved E-box binding sites within the ~2 kb promoter regions of *OsKO1* and *OsKO2*, respectively (Figures S4 and S5). We hypothesize that typical bHLH TFs, interacting partners of OsbHLH073, may bind to some of these sites to negatively regulate the expression of *OsKO1* and *OsKO2* in order to modulate GA homeostasis and cell elongation.

## 4. Materials and Methods

### 4.1. Plant Growth

T2 seeds of *osbhlh073-D*, o*sbhlh073-1, osbhlh074-1* (T-DNA insertional mutant, PFG_4A-02508, PFG_3A-17056, PFG_5A-00405), and wild-type (*Oryza sativa* cv. Japonica Dongjin) were germinated on half strength (1/2) Murashige and Skoog (MS) medium including 0.4% phytagel and 3% sucrose [70]. Plants were grown in the greenhouse and then transplanted into a field at Kyungpook National University (36^o^ N). PCRs for genotyping were conducted as explained previously [71,72]. The PCR primers are described in Table S1.

### 4.2. Hormone Treatment Analysis

T2 seeds of *osbhlh073-D* and wild-type were germinated in 1/2 MS medium in the light condition without or with 1 µM of GA_3_ added to the medium. For second leaf sheath analysis, seeds of o*sbhlh073-D* and Dongjin wild-type were grown into 1/2 MS containing various concentrations of GA_3_ under continuous light at 30 °C for seven days.

### 4.3. Vector Construction and Rice Transformation

The full-length *OsbHLH073* cDNA was amplified by PCR using the primer sets listed in Table S1. The *OsbHLH073* cDNA was cloned into the binary vector pGA3428 or pGA3438 [73], which contained the maize (*Zea mays*) ubiquitin (*GRMZM2G409726*) promoter. To generate the transgenic plants, *Agrobacterium tumefaciens* LBA4404 harboring the pGA3428 or pGA3438 was used for rice transformation [74].

### 4.4. RNA Isolation, RT-PCR, and Quantitative RT-PCR Analyses

Total RNA was extracted from various organs (seedling shoot, root, stem, young panicles, and internode) using QIAzol lysis reagent following by the manufacturer’s manual (Qiagen; https://www.qiagen.com). For cDNA synthesis, 2 µg of total RNA, reverse transcriptase (Promega, Madison, WI, USA), 10 ng of the oligo (dT) primers, and 2.5 mM of dNTP were used. Synthesized cDNAs were used as templates for RT-PCR and quantitative RT-PCR (qRT-PCR). SYBR green premix (Enzynomics, Daejeon, KOREA) and the Bio-rad instrument system (Bio-rad, USA) were used for qRT-PCR. Rice *actin 1* was used as an internal control. At least three biological replicates used for experiments and data were analyzed by Student’s *t*-test. The comparative *C*_t_ (2^−∆∆*C*t^) method was used to calculate the change of relative gene expression [74]. All primer sets are presented in Table S1 in this study.

### 4.5. Localization Assay and Transcriptional Activation Assay in Yeast

To investigate the cellular localization, we cloned an OsbHLH073-green fluorescent protein (GFP) fusion vector under the control of the maize ubiquitin promoter using pGA3452 [73]. The nuclear localization signal-tagged monomeric red fluorescent protein (NLS-mRFP) vector under the control of the *cauliflower mosaic virus (CaMV)* 35S promoter was used as marker to control localization in the nucleus. The OsbHLH073-GFP vector and the nuclear marker were transfected into the rice Oc suspension protoplasts using an electroporation method [75]. Protoplasts from Oc suspension cells were isolated as described previously [76,77]. Expression of the OsbHLH073-GFP fusion protein was monitored using a fluorescence microscope (Zeiss, Germany). To test the transcriptional activation assay, the *OsbHLH073* full-length ORF (Open Reading Frame) was cloned in-frame in the pBD-GAL4 Cam vector (Stratagene, La Jolla, CA, USA) to generate a BD:OsBHLH073 construct and the pAD-GAL4 vector (Stratagene, La Jolla, CA, USA) was used for AD:empty. An X-gal filter assay was conducted as explained previously [41].

## Supplementary Materials

The following are available online at https://www.mdpi.com/2223-7747/9/4/547/s1, Figure S1; Multiple sequence alignment of bHLH transcription factors, Figure S2; Phylogenetic tree of atypical and typical bHLH transcription factors, Figure S3; Multiple sequence alignment of the OsbHLH072, OsbHLH073, and OsbHLH074 proteins, Figure S4; Sequence of the *OsKO1* promoter region from the start codon, Figure S5; Sequence of the *OsKO2* promoter region from the start codon, Table S1: Primers used in this study.
