# Supplementary material for: OsbHLH073 Negatively Regulates Internode Elongation and Plant Height by Modulating GA Homeostasis in Rice
Source: Plants (Basel). 2020 Apr 23;9(4):547. doi: 10.3390/plants9040547 (PMC7238965; doi:10.3390/plants9040547)
Supplement: Supplementary file 1 [file plants-09-00547-s001.pdf]

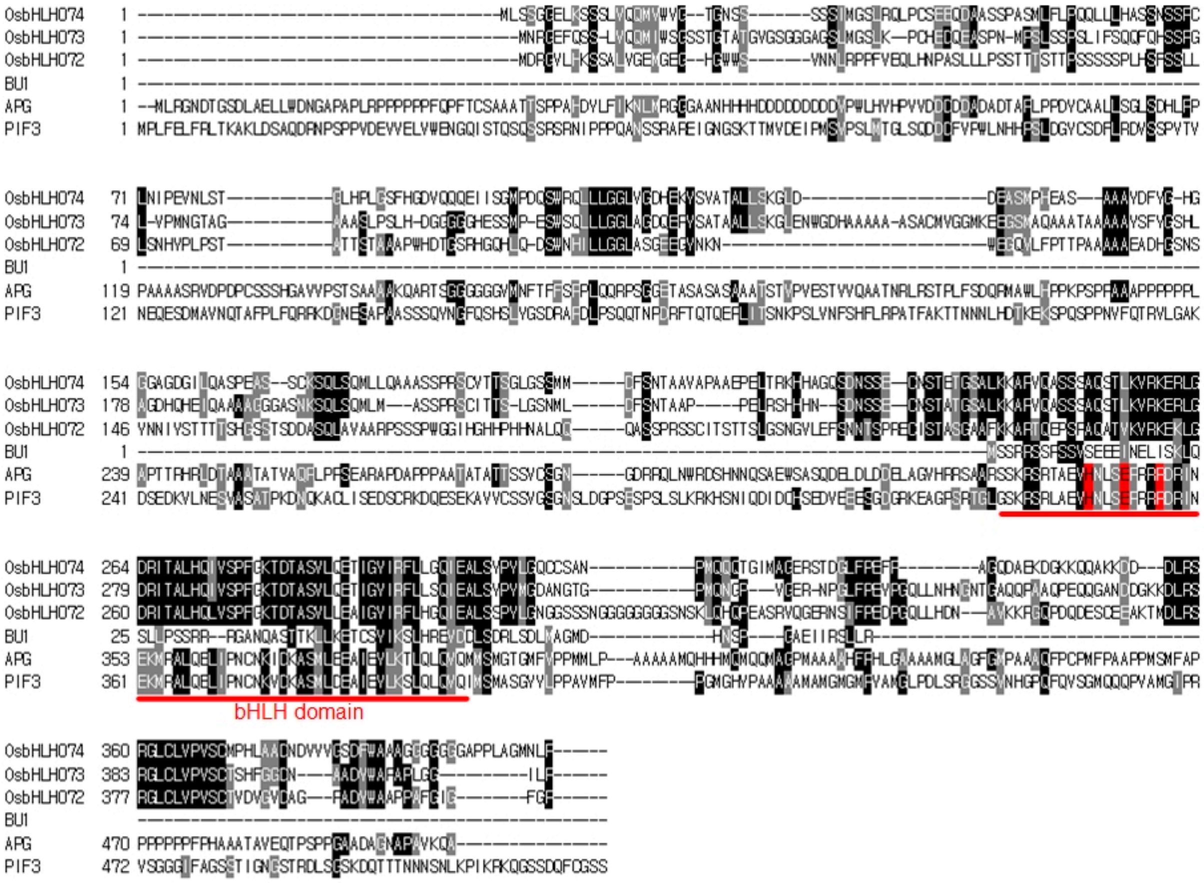

Figure S1. Multiple sequence alignment of bHLH transcription factors. The red shades refer to conserved amino acid sequence found in the typical bHLH domains. Underline indicates bHLH domain.

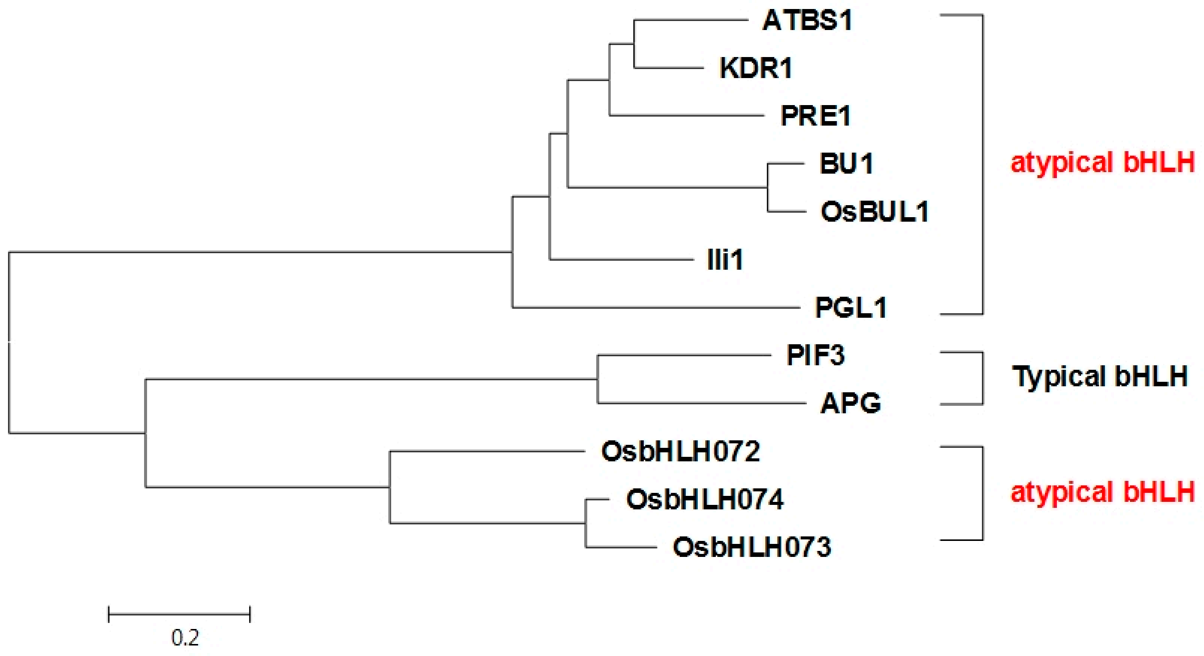

Figure S2. Phylogenetic tree of atypical and typical bHLH transcription factors from rice and Arabidopsis. Full-length amino acid residues were used from ATBS1 (AT1g74500), KDR1 (At1g26945), PRE1 (At5g39860), BU1 (Os06g12210), OsBUL1 (Os02g51320), Ili1 (Os04g54900), PGL1 (Os03g07510), PIF3 (AT1G09530) APG (Os05g04740), OsbHLH073 (Os05g14010), OsbHLH074 (Os01g13000), and OsbHLH072 (Os02g17680). Phylogenetic analyses were conducted with ClustalW and MEGA 7.0. Tree was generated by Neighbor-joining method.

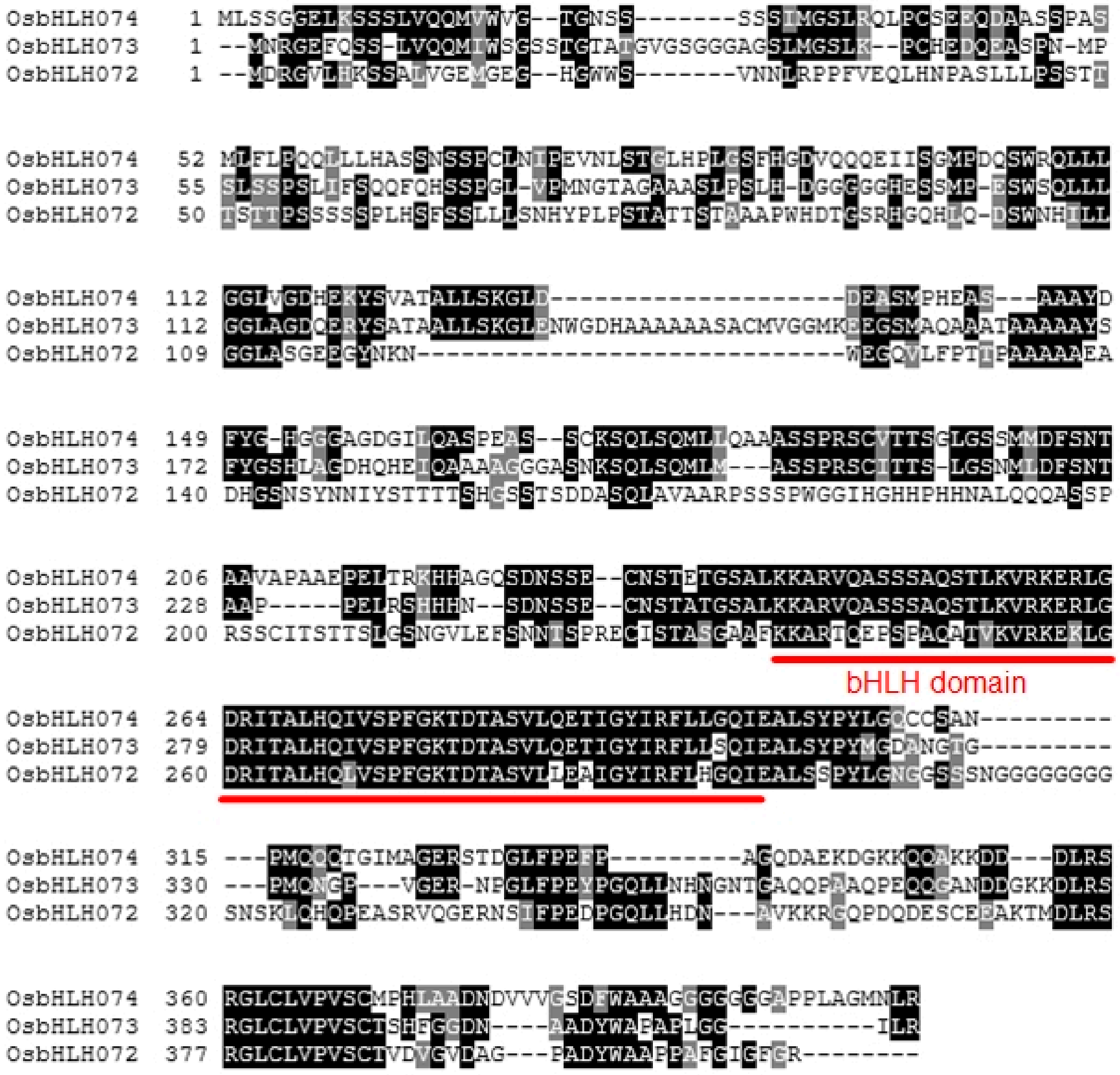

Figure S3. Multiple sequence alignment of OsbHLH072, OsbHLH073, and OsbHLH074 proteins. Black shade indicates identical amino acid and gray shade indicates similar amino acids. Underline indicates bHLH domain.

>OsKO1 (LOC_Os06g37224) promoter region (~2 kb from ATG)

catttttgggataagtttttctaggggtctacttgcgaaataagtttttcaaaaggactaaaatgtgaaaaatc**cagatg**
gtctctaatcattaatgtattcaaatactttttcttactcttctttccttcctttcaccaccatgcaacaaaatttactctaata
aatgctaagagtcaactctgagccgttgtcatgcataacaaccatacttctttctctttcttccatatcatcaaatttactt
gtatgacaatagagagagaccactaataaataccattgtacatgctctagttgcctgggaagacagttaatcggtgg
attttaccgcatacaaatcgctgcgccttgatgggtcgagcgcttgacacccgggcagccgcgtgctgccccagg
aggtaaatcctccactgatccctgctcgtattataagatgtttgatttttttttcctaatcaaactttcttaagtttcatcaaa
tttataaaaaaatttaacagcatacactagtggagaaatgctttgtagtcccggttggtaacatcctgtagtcccggttt
tttaaccgggactacgaatctgggactaaagatcgctatctttagtcccggttcaaatactcaggactaaaaatatgg
tcc**cagttg**ttgttaccgaccgggactttttttcttttcttttcatccggttggtatttttcctttttttttttctttcgcccaaatct
tcgcggatcgaacgaataaaatcaacaagaaagcctaaatcatagatcgaataaataaaatcaacaacaaagcat
ccatattcttttacacttattcacatctacaacaaagcatccatgttcttttacacttattcacagtacaacatcttcagaa
caacgagcattcattacagagaatagagaaagaaaaaaaaagttcacatctacaacaacatcttcagcattcatcag
ataacacagagaagagaagaaaaaaaaaatgttcgtccgccagccgccgcctcctgaccggcgccggcctccat
cccgccgtcccgccggaggggaagggaggggaggtgggggaggagaggggaggaggagatggggtgag
agaagaaggaaggggaggatgggaggagaggagagaagaagataagatgggggatggctggatgggagag
gaggagataaggatgggaggatagaggcttttttttttacccgttgtttaacaaccgggactaaaaatagatctttgg
tcccggttggtgttactaactgggactaaagatcaccgtgatctttggtcccggttggtaatatcaaccgggacaaa
agttaggttactaaccgagactaaaaaccccaggagggcctgacagcccatgaaaacatgcaaaccaggactaa
aaatcatctttagtctcggttggtgtcacatagtccctttgggcttataaaccggaactaaaaattatttttagtcccggt
ttttattggaaccgggactattgtggaatttggtcgaccgaccaaagatgatttctccgatagtgatagaaaatataaa
ttggttttaataaattgaacattaaatatatttttataatatgtttctttttaatattactatattttttttataaatttagtcaatttaa
ataagtttgactagtaaaatactagctagtcaaacaactacatgcatcgtgcatggatattacttcccgacgacaact
attgtaaaagtctgatcttaagttcaaac**cacgtg**agctgaccgcatgatcaacgcattatgcggataccttaattaa
gttcccaagtcaatcaaattgagggtacaagacgaaattgattcccccacacacttgctccttcttccacttccatcct
gccctatataatatatatatatatatatatatatatatatatatatatatatatatacttagtactgtacgtactaaaccgggga
ttgatcaggagagtgcagtgcaaactcgcagtgaacctgtgtgatcgatcggcc**ATG**

Figure S4. Information of *OsKO1* promoter region from start codon (ATG). We manually found E-boxes in the promoter regions using conserved nucleotide sequences. Red letter indicate predicted E-box sites.

> OsKO2 (LOC_Os06g37300) promoter region (~2 kb from ATG)

attggctaagggtgtgtttagttcgcgaaaaggatgattagaaggaaatatatatcctatgtacataaacttctcgtac
acactccatgcatatcaaatagcaaatgccatagaaaattctagaaaaaatcggacaaatactaaaaatcttagttct
ctgattggaaatttatgttgcgaagtttttttttcactttttaagacgccagtactgattatgaataaataaaagaaaagg
gacatcatctgctatcccactatcatatcggagttgagagagcggagataaagaaaggatggagatggtcatcagt
gtcattcctcccacttcgggacggtgacgaggaagcgctcaagcggtgccaaatccgatggccaaggatctcgg
ggtttcaaatccaccacggacaagcaacgaagacaagtaacggagtttcaaattcccccatattgtgtgaactatga
agttctgattttgatcgcaatgaccgcttcctccctcgccatgaccctaggcaagaggaggaggaggaccaggaa
actttatcgatactatgctattagccattaatacgagcacatgtgccacgtctaaaacttgaatttgggtaggcaagttt
caccataagaaatctaaacaattgagttatgctcactttacagttatataacttttaattaacccgttccaatggtcatat
cttagttttattttgaatggcgtagttcctatataatattattataaaatatatgtgttttctattcggtattttagtctgcatatt
gttgtcaggaaggagataaggatttgtcacataacttcgtatctttttattatctttttaaaattcttgaccgtgtttatattg
acagagatactgtttttattattaaaaattgatcgggccttaatgaaaactatcatggtcggactaatttataacttctgtg
tttattttttaaggcaaactaatatggtcgacactagataaagaactgacgtaaattctttgagtgaaaactttcaaatat
aactactatagtgtaattccaatataactatactataactacactaaaaattttatgtaacttgtatatatatataagtattaaa
atgcatgtcactttatagtaagttatatatatataaaggtatatagttatagtgtaattacactgtcgtgatatcaatattac
accacagttacattgaaaaatcttttaagtcgatatttagcaattaccaagtgggtattaataacgaggagaacagca
aaagtaattagtcgaaactcgaaaatcaggtgtacttgcatatgggtattacttggcataaaggctggtcttaatttca
aagcacgtgagctgaccgcgcgatgaatacctacctactacatgtggctcccaaggagtacagaagaaattaaac
gcaaattaatgaatgtgcaagcgtatgtgtacatggccctaccaaacaatgacgacaacgacactacaagaacga
cgtaatatttttaattgaggtagctgctacacgtgttaaatttgtattacaaagctcttaaaaaattaatgatatattcacat
cgtgaaacaaagtgatgttaagactttattttttttaagaatagtacgtatagacttattattctagtgtgtttgaaagaa
gaaagaagattaagaaaataaaccattaaatatgattaattaagtattaattattttaaacataaaaaacaaattagtgt
aatttatttatagaaaaaattataaaagaaaaatgatgattactactccctccgtccaataaaaaatctatcagaggttg
tttaagggagtactcacgtggaaagggagcgaaacctctctctttcttctagcaacaaacggtcgaacgcagccaa
aaagcaacaaaaaagacttcaagttggtacagcgacgacgccgccctttcttatttcctcgctcttcgacgtgctgc
ggcctctataaataagaaagccgggattaattaaagagtgcgtgtgatcagatcgtgtgcc**ATG**

Figure S5. Information of *OsKO2* promoter region from start codon (ATG). We manually found E-boxes in the promoter regions using conserved nucleotide sequences. Red letter indicate predicted E-box sites.

Table S1. Primers used in this study

Name	Primer sequence (5′→3′)	Remarks
CPS1-qF	GCGTGCATTTTCGAACCAA	qPCR
CPS1-qR	TTGGCCAGCACTGACACTCT	qPCR
GA20ox1-qF	GCCACTACAGGGCCGACAT	qPCR
GA20ox1-qR	TGGTTGCAGGTGACGATGAT	qPCR
GA20ox2-qF	CCAATTTTGGACCCTACCGC	qPCR
GA20ox2-qR	GAGAGAAGCCCAACCCAACC	qPCR
GA3ox2-qF	TCCTCCTTCTTCTCCAAGCTCAT	qPCR
GA3ox2-qR	GAAACTCCTCCATCACGTCACA	qPCR
GA2ox1-qF	TGACGATGATGACAGCGACAA	qPCR
GA2ox1-qR	CCATAGGCATCGTCTGCAATT	qPCR
GA2ox3-qF	TGGTGGCCAACAGCCTAAAG	qPCR
GA2ox3-qR	TGGTGCAATCCTCTGTGCTAAC	qPCR
Slr1-qF	CTTCGAGCCCGTGCACCTGG	qPCR
Slr1-qR	CCGTAAGCCCGATCGGTCGC	qPCR
KO1-qF	AGTAGCCAAGGAGGCGATGA	qPCR
KO1-qR	CGGCTTATCACAGACAATGCT	qPCR
KO2-qF	TGAAGTAGCCAAGGAGGCGA	qPCR
KO2-qR	CGCTGATTGCGACCATACTTT	qPCR
OsbHLH073-qF3	CCTCCTCTTCAGCACAATCTAC	qPCR
OsbHLH073-qR3	GCCAATGGTCTCTTGCAGTA	qPCR
Actin-qF	CAGCCACACTGTCCCCATCTA	qPCR
Actin-qR	AGCAAGGTCGAGACGAAGGA	qPCR
OsbHLH073-OE-F1	GTTAACatgaatagaggagaattccagagctc	Overexpression, Localization
OsbHLH073-OE-R1	ACTAGTGGTACCccgtaggatcccgccgag	Overexpression, Localization
4A-02508-F	TTACCCCTAGGTACAGGGGTG	*OsbHLH073-D* genotyping
4A-02508-R	TACTGTAATAAGCAAGCCGGC	*OsbHLH073-D* genotyping
LB	CTAGAGTCGAGAATTCAGTACA	T-DNA border for genotyping
3A-17056-F	GCGTCTGTACTGCAAGAGACC	*OsbHLH073* KO-Genotyping
3A-17056-R	AATACCGGATGTGACACATTC	*OsbHLH073* KO-Genotyping
5A-00405-F	GAGCAACTGGTCCTCCCTG	*OsbHLH074* KO-Genotyping
5A-00405-R	CAATTTGCAAAACGCCTCTAC	*OsbHLH074* KO-Genotyping
OsbHLH073-RT-F1	CCTCCTCTTCAGCACAATCTAC	RT-PCR for *OsbHLH073*
OsbHLH073-RT-F1	GTAAGGTTGATTGCTCTTGATGC	RT-PCR for *OsbHLH073*
OsbHLH074-RT-F1	CATGCAGGCCAGTCAGATAA	RT-PCR for *OsbHLH074*
OsbHLH074-RT-F1	TCAGGTCGTCGTCCTTCTTC	RT-PCR for *OsbHLH074*
Actin-RT-F1	AGGCTCCTCTCAACCCCAAGGCCAATCG	RT-PCR for *OsActin*

Actin-RT-R1	AGGTAATCAGTGAGATCACGCCCAGC	RT-PCR for *OsActin*
